# Alcohol consumption during pregnancy: the result of a risky consumption trajectory?

**DOI:** 10.1590/0102-311XEN129523

**Published:** 2023-08-07

**Authors:** Claudia de Souza Lopes

**Affiliations:** 1 Instituto de Medicina Social, Universidade do Estado do Rio de Janeiro, Rio de Janeiro, Brasil.

The article by Cabral et al. ^1^, published in this issue of CSP, addresses a public health problem in Brazil and worldwide: the high prevalence of alcohol consumption during pregnancy and its effect on the health of the mother and fetus.

Alcohol consumption during pregnancy is associated with many gestational outcomes, including stillbirth, miscarriage, preterm delivery, intrauterine growth retardation and low birth weight ^2^ ^,^ ^3^ ^,^ ^4^, and a variety of lifelong conditions known as fetal alcohol spectrum disorders (FASDs) ^5^ ^,^ ^6^. One of the most disabling potential outcomes of alcohol consumption during pregnancy is the risk of developing fetal alcohol syndrome (FAS), the most severe and visibly identifiable FASD, which causes permanent brain damage, congenital anomalies, and deficits in cognitive behavioral, emotional, and adaptive functioning ^7^.

To mitigate the problem, for more than a decade, guidelines from international organizations/institutions have recommended that women who are pregnant or planning to become pregnant abstain from any alcohol consumption during this period ^8^ ^,^ ^9^.

Despite this, the prevalence of this consumption during pregnancy remains high worldwide. However, Brazil has a gap on this topic, with few studies and most of them conducted in specific populations. The article by Cabral et al. ^1^ fills this gap as the first nationally representative population study to evaluate the set of factors related to the Brazilian reality of inequalities in access to services and social vulnerability that contribute to the high prevalence of alcohol consumption among pregnant women. Among the factors associated with higher prevalence of alcohol consumption during pregnancy and the presumptive diagnosis of misuse, young age at pregnancy stands out, especially among adolescents aged 12 to 19 years. This is one of the most important indicators of social vulnerability, causing psychosocial and economic harm in women’s lives.

Thus, understanding the role of early alcohol consumption in this scenario will allow the development of public policies aimed at greater surveillance of alcohol consumption in this age group and the screening of groups at higher risk for alcohol consumption during pregnancy. To what extent does the high prevalence of alcohol consumption among women in adolescence and early adulthood affect its persistence during pregnancy?

To answer this question, we need to take a step back and consider trends in alcohol consumption among adolescents and young adults in recent decades.

Studies have shown that, despite a downward trend in the prevalence of alcohol consumption among women (a trend also observed among men), binge drinking has grown among young women of reproductive age worldwide ^9^ ^,^ ^10^ ^,^ ^11^. However, these findings are quite heterogeneous when considering different regions of the world, age groups, and drinking patterns.

Data from two editions of the *Global Status Report on Alcohol and Health* ^8^ ^,^ ^9^ show that, from 2010 to 2016, despite the reduction in overall alcohol consumption among women aged 15 years and older in almost all countries in Latin America, this was not the case in Brazil, where rates remained the same (8.9% for both periods). Moreover, prevalence rates of heavy episodic drinking (≥ 60ml of pure alcohol at least once in a month) among women aged 15 years and older who had ever consumed alcohol in their lives increased in almost all countries in Latin America, with rates ranging from 0.1% in Chile to 11.1% in Brazil in 2010 and from 14.7% in Chile to 27.4% in Peru in 2016 (in Brazil, this rate was 25.1%). The only exceptions were Venezuela and Paraguay, with rates of 21.8% and 41%, respectively, in 2010, which decreased to 18.2% and 24.6%, respectively, in 2016. Most of these rates are significantly higher than the overall prevalence of heavy episodic drinking among women, which was 19.9% in 2016. These data show that Latin American women not only drink at high rates, but many of them are involved in risky patterns of alcohol consumption. Thus, some women are likely to continue drinking during pregnancy or before they know they are pregnant. Moreover, a study showed that Latin America and the Caribbean have the highest proportion of unintended pregnancies (56%), while in other continents (Africa, Asia, Europe, North America, and Oceania) these rates range from 35% to 51%, within a world average of 40% ^12^. Coupled with the relatively high rates of alcohol consumption and risky drinking patterns, this may lead to a higher risk of alcohol-exposed pregnancy in these countries.

In Brazil, population studies have pointed to high alcohol consumption among women, with an increase also among adolescents.

The study by Caetano et al. ^13^ with data from the *Brazilian National Survey of Alcohol and Drugs* showed an increase among both men and women in consumption per week (men: 12.82 in 2006, 15.78 in 2012, p < 0.01; women: 4.89 in 2006, 7.66 in 2012, p < 0.001) and in the proportion of excessive alcohol consumption (men: 57% in 2006, 66% in 2012, p < 0,05; women: 39% in 2006, 48% in 2012, p < 0.05), although this did not occur in all genders and age groups.

The *Study of Cardiovascular Risk Factors in Adolescents* (ERICA), a Brazilian school-based study that evaluated 74,589 adolescents, observed a similar prevalence of alcohol consumption in the last 30 days for boys (21%) and girls (21.5%), with higher among adolescents aged 15 to 17 years (29.3%) ^14^.

A study based on data from 100,914 ninth-grade students from the 2015 *Brazilian National Survey of School Health* (PeNSE) observed that girls were more likely to try alcohol (OR = 1.09; 95%CI: 1.05-1.12) and to have consumed it in the last 30 days (OR = 1.09; 95%CI: 1.00-1.13) compared with boys. PeNSE 2015 also showed that regular alcohol consumption, despite its very high prevalence, especially among girls, decreased from 27.3% (2009) to 23.2% (2015) ^15^.

In a recent study conducted in the 2004 Pelotas (Brazil) birth cohort, the prevalence of alcohol and cigarette experimentation increased at age 15 among girls ^16^.

Studies conducted in different regions of the world show that high alcohol consumption before pregnancy is one of the most important risk factors for persistent consumption during pregnancy ^17^ ^,^ ^18^ ^,^ ^19^. Moreover, many pregnant women may consume alcohol before they discover their pregnancy and maintain their usual pattern of alcohol consumption in the early weeks of an unplanned pregnancy ^20^ ^,^ ^21^. A recent study conducted in Canada from five cohort studies of pregnant women pointed that, after adjustment for multiple risk factors, women’s alcohol consumption during pregnancy - both normal consumption and binge drinking - was related to alcohol consumption before pregnancy ^22^.

Other important studies sought to assess risk behaviors among adolescents and young people, the concomitance of these behaviors, the most vulnerable groups, and the effect of such behaviors on adulthood, including the persistence of heavy alcohol consumption ^23^ ^,^ ^24^.

According to the 2018 *Global Status Report on Alcohol and Health* ^9^, alcohol consumption, especially by adolescents and young women, is associated with unprotected sex ^25^ ^,^ ^26^ and increases the risk of unwanted pregnancy ^27^ ^,^ ^28^ ^,^ ^29^ and the risk of fetal exposure to alcohol due to delayed pregnancy discovery ^27^, with negative implications for newborns ^30^. In Brazil, a study with data from the 2019 *Brazilian National Health Survey* (PNS) showed that heavy alcohol consumption among women aged 18 to 24 who were single/not cohabiting was associated with inconsistent condom use ^31^.

Studies also pointed that early age of onset of alcohol consumption is associated with increased early initiation of sexual intercourse and early pregnancy ^32^, favoring the persistence of alcohol consumption during pregnancy ^33^. According to another study, maternal age at first birth was associated with high risks: younger mothers were more likely to have a history of high-risk drinking compared with older mothers ^34^.

Therefore, knowing the scale of the problem and better understanding the groups most at risk will allow the establishment of public policies aimed at surveillance during prenatal care and developing screening strategies to increase access to health services for pregnant women.

Promoting studies that seek to understand the factors involved in alcohol consumption by adolescents and young people - especially early age and heavy drinking among girls - should be the initial step for the implementation of public policies aimed at preventing this consumption and counseling on alcohol consumption by women of reproductive age. This consumption, as aforementioned, affects not only the health of adolescents and young people in the short term, but when it coexists with conditions of socioeconomic vulnerability, it often persists, increasing the risk of alcohol consumption during pregnancy. Thus, studies aimed at assessing the prevalence of alcohol during pregnancy and seeking to identify the most vulnerable groups, such as the study by Cabral et al. ^1^, are an important step. Periodic nationally representative population surveys that include adolescents and adults are also needed to assess trends in alcohol consumption. Moreover, cohort studies that allow the follow-up of adolescents into adulthood have the ideal conditions for analyzing the trajectory of these disorders throughout life and the main risk factors involved in the initiation and persistence of alcohol consumption.
